# Effects of Metabolites Derived From Gut Microbiota and Hosts on Pathogens

**DOI:** 10.3389/fcimb.2018.00314

**Published:** 2018-09-14

**Authors:** Zhendong Li, Guomei Quan, Xinyi Jiang, Yang Yang, Xueyan Ding, Dong Zhang, Xiuqing Wang, Philip R. Hardwidge, Wenkai Ren, Guoqiang Zhu

**Affiliations:** ^1^Joint International Research Laboratory of Agriculture and Agri-Product Safety of Ministry of Education of China, Jiangsu Co-innovation Center for Important Animal Infectious Diseases and Zoonoses, College of Veterinary Medicine Yangzhou University, Yangzhou, China; ^2^College of Veterinary Medicine, Kansas State University Manhattan, KS, United States; ^3^Guangdong Provincial Key Laboratory of Animal Nutrition Control, Subtropical Institute of Animal Nutrition and Feed, College of Animal Science, South China Agricultural University Guangzhou, Guangdong, China

**Keywords:** metabolites, pathogen infection, gut microbiota, dietary nutrients, regulatory effects

## Abstract

Intestinal metabolites participate in various physiological processes, including energy metabolism, cell-to-cell communication, and host immunity. These metabolites mainly originate from gut microbiota and hosts. Although many host metabolites are dominant in intestines, such as free fatty acids, amino acids and vitamins, the metabolites derived from gut microbiota are also essential for intestinal homeostasis. In addition, some metabolites are only generated and released by gut microbiota, such as bacteriocins, short-chain fatty acids, and quorum-sensing autoinducers. In this review, we summarize recent studies regarding the crosstalk between pathogens and metabolites from different sources, including the influence on bacterial development and the activation/inhibition of immune responses of hosts. All of these functions would affect the colonization of and infection by pathogens. This review provides clear ideas and directions for further exploring the regulatory mechanisms and effects of metabolites on pathogens.

## Introduction

A growing number of studies suggest that intestinal microbiota play an important role at the interface of health and disease of hosts, due to a crosstalk of small molecules between the intestinal mucosal surfaces and the microbiota. Examples include food digestion, oxidation and reduction of molecules, and the synthesis of essential amino acids. Some of these molecules are metabolites derived from hosts and microbiota (Donia and Fischbach, 2015). Many studies have reported that the intestinal metabolites regulate pathogen infection in intestines, through genome-based analysis of bacteria and high-throughput metabolomics (Browne et al., 2017; Hirata and Kunisawa, 2017).

Metabolites can affect the invasion of pathogens through different pathways. Some metabolites, such as nisin, and several lantibiotics directly kill pathogens by disrupting bacterial cell structures. Nisin inhibits peptidoglycan synthesis by targeting lipid II, which is a key intermediate in the peptidoglycan biosynthesis machinery within the bacterial cell envelope. Lantibiotics dock with lipid II to promote the formation of pores in the cell membrane, resulting in a loss of membrane and subsequent cell death. In addition, bacteriocins can kill bacterial cells by interfering with bacterial DNA, RNA, and protein metabolism (Cotter et al., 2013). Resource competition between commensal bacteria and pathogens caused by metabolites is also an indirect pathway. Competition in the use of monosaccharides can limit the colonization of *Citrobacter rodentium* in wild-type mice (Kamada et al., 2012). Another example is iron that is crucial in host–pathogen interaction, in which both opponents compete for iron. Pathogens have specific iron requirements due to the metal's function in metabolism and proliferation (Nairz et al., 2010). Several metabolites also affect cell adhesion and biofilm formation. For example, D-amino acids produced by *Bacillus subtilis* prevent biofilm formation and reduce existing biofilms (Mühlen and Dersch, 2016). Quorum-sensing autoinducers also affect the adhesion of flagellum, biofilm formation, and production of toxins (Yang et al., 2013). Recent studies have uncovered a major role of metabolites in the regulation of the immune system (especially the microbial metabolites). Intestinal metabolites activate innate immunity through numerous microbial sensors called pattern recognition receptors (PRRs) in intestinal epithelial cells after infection by pathogens. In addition, several microbial metabolites are involved in the regulation of adaptive immune cell development, in particular T lymphocytes (Levy et al., 2017). Furthermore, some of these identified metabolites, including short-chain fatty acids (SCFAs) and vitamins, have been shown to have a protective effect, whereas others, such as quorum-sensing autoinducers, have been shown to directly promote the infection by pathogens.

This review summarizes the findings from past studies on the effects and brief regulatory mechanisms of metabolites from different sources on pathogens in intestines. The growing understanding of the role of intestinal metabolites in bacterial virulence provides new opportunities to interfere with important pathogens or pathogenesis.

## The effects of metabolites from gut microbiota on pathogens

Gut microbiota is mainly metabolized via absorbing energy and sources from the host and the diet to support their growth and releasing metabolites produced through fermentation. These metabolites in turn influence the host metabolome and the formation of gut microbiota (Flint et al., 2015). Here, we describe the sources of several intestinal microbial metabolites (Table 1), explain in detail how these microbial metabolites regulate pathogens and their effects, and provide a future research direction. (Table 2).

**Table 1:** The evidence of metabolites from gut microbiota.

**Metabolites**		**Gut Microbiota**	**References**
Bacteriocin		All major lineages of Bacteria, Archaea.	Gillor et al., 2008
	Nisin A	*L. lactis*	Hurst, 1981
	MccB17	*Enterobacteria* (mostly *E. coli*)	Li et al., 1996
	MccJ25	*Enterobacteria* (mostly *E. coli*)	Bayro et al., 2003
	Colicin	*E. coli*	Cascales et al., 2007
SCFA	Acetate Propionate	*Bacteroidetes*	Macfarlane and Macfarlane, 2003
	Butyrate	*Firmicutes*	Macfarlane and Macfarlane, 2003
Microbial amino acids	Lysine	Gut microbiota	Metges et al., 2003
	D-aas	Gut microbiota	Sasabe et al., 2016
Microbial Vitamins	Vitamin K2, B vitamins	LAB, *Bifidobacteria*	Hill, 1997
AI	AI-2	*E. coli* and other bacteria	Pereira et al., 2013
	AHL	*P. aeruginosa, Clostridium perfringens*	Yang et al., 2014
	PQS	*P. aeruginosa*	Diggle et al., 2006

**Table 2:** The pathogens are regulated by gut microbiota-derived metabolites and their mechanisms and effects.

**Metabolites**		**Pathogens**	**Mechanisms and effects**
Bacteriocin	Nisin A	*S. enterica, S. aureus, B. cereus*.	Bind with peptidoglycan precursor lipid II to prevent the synthesis of peptidoglycan. Induces pore formation. ↓
		*S. enterica, S. aureus, B. cereus*	Synergistic effects between the L. acidophilus surface layer and Nisin A. ↓
	MccB17, MccJ25	*Escherichia, Klebsiella, Shigella, Salmonella, and Pseudomonas*	Inhibit the bacterial RNA polymerase and DNA gyrase. ↓
	Colicin	*E. coli*	Split the 16S rRNA at the 3′end of the coding sequence and inhibit protein synthesis. ↓
SCFA		*C. jejuni*	Induce Caco-2 cell differentiation. ↓
			Active AMPK and mucin secretion. ↓
		*S. aureus*	Govern the expression of antimicrobial peptide mRNA and enhance the integrity of BMEC. ↓
		All pathogens	High concentrations of SCFAs release anions and then affect osmotic balance of bacteria. ↓
		EHEC strain	Low concentrations of butyrate obviously promote the expression of vital virulence. ↑
		*C. jejuni*	Different concentrations of SCFAs can be used as spatial clues to permit colonization and gene expression of pathogens. ↑
Microbial amino acids	D-aas	*E. coli*	Replace the D-Ala in short peptide of peptidoglycan and result in the accumulation of two major new muropeptides, which affect cell walls. ↓
		*S. aureus*	Affect biofilm formation and degradation. ↓
		*V. cholera, P. aeruginosa, S. aureus*, EHEC	DAO deaminates neutral D-aas to release antimicrobial product H_2_O_2._ ↓
	D-Ser	EHEC	Activate the SOS response and inhibit the expression of the type III secretion system. ↓
Vitamins	Vitamin K2	*S. aureus*	Participate in a redox cycle with heme on membrane, and then keep development of pathogens. ↑
		*S. aureus*	Enhance biofilm formation. ↑
	menadione	Gram-positive organisms	Inhibit bacterial growth and exotoxin. ↓
	Vitamin B2	Many pathogens	Stimulate proliferation of neutrophils, monocytes, and macrophages. ↓
	Vitamin B9	*L. monocytogenes*	Activate Nox2 and keep defense against pathogens. ↓
	Vitamin B6	*S. typhimurium*	Regulate lymphocyte proliferation, natural killer cell activity, and Treg cells, Favor the development of cellular and humoral immunity. ↓
Autoinducers	AI-2	*E. coli*	Lack of AI-2 reduces the adhesion of flagellum, expression of Stx2e toxin, and production of bacterial surface outer membrane proteins. ↓
	AHL	*S. aureus* F18 *E. coli* *P. aeruginosa*	AI-2 negatively regulates biofilm formation and higher polysaccharide intercellular adhesion (PIA) production. ↓ Enhance the expression of flagella, a virulence factor. ↑ Activate target genes including those coding for rhamnolipid, elastase, the PA-IL lectin, and pyocyanin and influence biofilm formation. ↑

*↓, Inhibit pathogens; ↑, Promote pathogens*.

### Bacteriocins

Bacteriocins were first identified in *Escherichia coli*. Fredericq discovered that bacteriocins are proteins and can be active against a limited range of bacteria, but have a specific immunity mechanism for their producers (Fredericq, 1946). Bacteriocins have been found in all major lineages of bacteria and some members of the archaeal community (Riley and Wertz, 2002; Nes et al., 2007; Blum, 2008). In particular, 90% of environmental and clinical isolates of *Pseudomonas aeruginosa* can produce bacteriocins. The bacteriocins of gram-positive bacteria are plentiful and even more diverse than those generated in gram-negative bacteria. The most typical bacteriocin-producing gram-positive bacteria are *lactic acid bacteria* (LAB) (Gillor et al., 2008).

**Figure 1:**
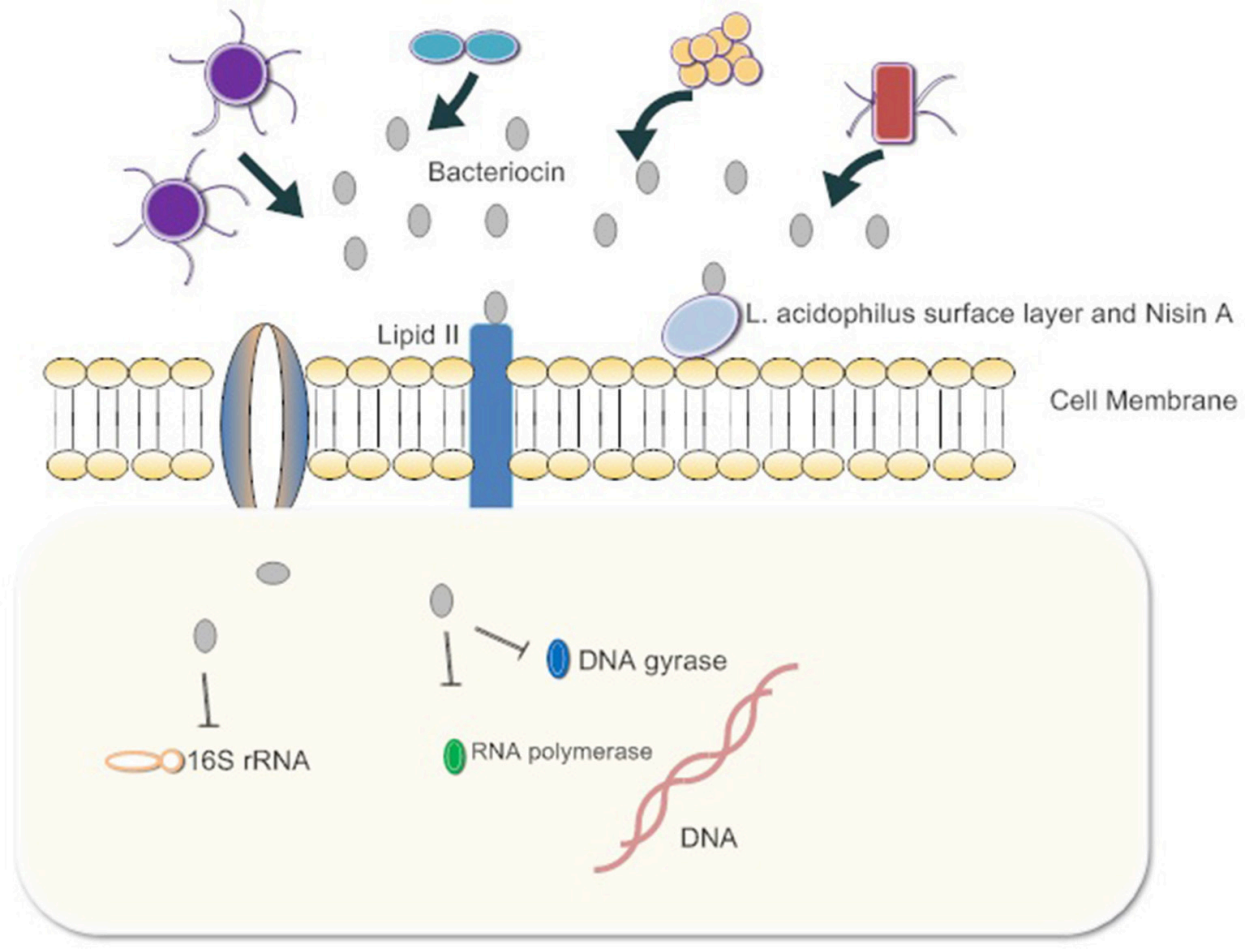
The gut microbiota release bacteriocins. First, they bind Lipid 2 of the cell wall of the pathogen, inhibiting the synthesis of peptidoglycan. Some bacteriocins enter the cytoplasm of pathogens and bind 16s rRNA to inhibit protein synthesis. Bacteria also inhibit RNA polymerase and DNA gyrase. Ultimately, cell growth is blocked or cells die.

The interactions of bacteriocins with target bacteria have been studied in many respects (Figure 1). Since the cell wall is highly conserved among pathogens and does not exist in mammalian cells, it is considered to be a perfect target for inhibiting pathogen infection. A suitable antibacterial substance can inhibit the formation of the cell wall or destroy its integrity (Mazzotta and Montville, 2010; Nayar et al., 2015). For example, nisin A, produced by *Lactococcus lactis*, is a typical poreforming bacteriocin which binds peptidoglycan precursor lipid II to prevent it from combining with penicillin. This combination induces pore formation by nisin molecules arranged as pore-forming “units” and then rapidly kills cells Lages et al., 2013). The synergistic effects of the *Lactobacillus acidophilus* surface layer and nisin A inhibit the growth of pathogenic gram-negative *Salmonella enterica* and potential pathogenic gram-positive bacteria, *Staphylococcus aureus and Bacillus cereus*. The effect suggests that the synergistic action between bacteriocins and beneficial bacteria can inhibit harmful bacteria. If this mechanism is ubiquitous in other intestinal commensal bacteria, exogenous bacteriocins can enhance the antibacterial capacity of commensal bacteria, and quickly clear the pathogens (Prado-Acosta et al., 2010). In addition, the genetic materials within pathogen cells are targets of bacteriocins. For example, the MccB17 and MccJ25 microcins are toxic peptides secreted by enterobacteria (mostly *E. coli*), with molecular masses below 10 kDa. Microcins are produced and secreted under conditions of limited essential nutrients and are activated against related species. MccB17 and MccJ25 microcins internalize into the target bacterial cell respectively across the outer-membrane protein OmpF and the outer-membrane receptor FhuA-dependent TonB pathway. The inner-membrane protein SbmA then transports both of the microcins across the inner membrane to the cytoplasmic face. Finally, inside the cell, MccJ25 inhibits the bacterial RNA polymerase and MccB17 acts as a DNA gyrase inhibitor (Mathavan and Beis, 2012). MccB17 exhibits potent bactericidal activity against a wide range of bacteria including *Escherichia, Klebsiella, Shigella, Salmonella, and Pseudomonas* (Baquero and Moreno, 1984). Besides, bacteriocins can inhibit the synthesis of key proteins in pathogens. The colicin family proteins by *E. coli* exhibits 16S rRNase activity. They bind the BtuB/Tol translocation machinery for the purpose of crossing the outer membrane and split the 16S rRNA at the 3-end of the coding sequence, inhibiting RNA translation (Toba et al., 1988; Lancaster et al., 2010; Ng et al., 2010).

Importantly, in animal models, bacteria have been found to produce bacteriocins and inhibit the growth of pathogens. For example, *E. coli* strain H22 inhibited many *Enterobacteriaceae* members *in vitro*, whereas *in vivo*, 6 days after simultaneous oral inoculation in germ-free mice, *E. coli* H22 decreased the fecal population of *Shigella flexneri* 4 to undetectable levels. Further studies have revealed that *E. coli* H22 produces a variety of bacteriocins to prevent pathogens, including microcin C7, colicin E1, and colicin Ib (Cursino et al., 2010).

Currently, most studies focus on the discovery of novel bacteriocins produced by bacteria and their functions *in vitro*. Isolating bacteriocins from the intestine and mimicking the intestinal environment to explore the types of bacteriocin production and the regulatory mechanisms for pathogens could be a useful research direction to better understand the interactions between gut microbiota, bacteriocins, and pathogens. More importantly, to address the problem of antibiotic resistance, using the synergy of bacteriocins and antibiotics is a new approach in the treatment of infectious diseases (Sass and Brötz-Oesterhelt, 2013).

### Microbiota-derived short-chain fatty acids

Short-chain fatty acids (SCFAs), the metabolites of microbial fermentation, consist of acetic acid, butyric acid, and propionic acid. Acetic acid and propionic acid are mainly produced by *Bacteroidetes* fermentation, whereas butyric acid is the main metabolite of *Firmicutes* (Macfarlane and Macfarlane, 2003). SCFAs are mainly observed in the cecum and the ascending colon, but less in the small intestine (Cummings et al., 1987). When nondigestible carbohydrates such as fiber reach the colon, they are hydrolyzed into oligosaccharides by anaerobic bacteria. Oligosaccharides are then converted to phosphoenolpyruvate (PEP) via two major bacterial metabolic pathways, the Embden–Meyerhof–Parnas pathway and the pentose phosphate pathway. Finally, PEP is transformed into an organic acid or alcohol (Fischbach and Sonnenburg, 2011).

**Figure 2:**
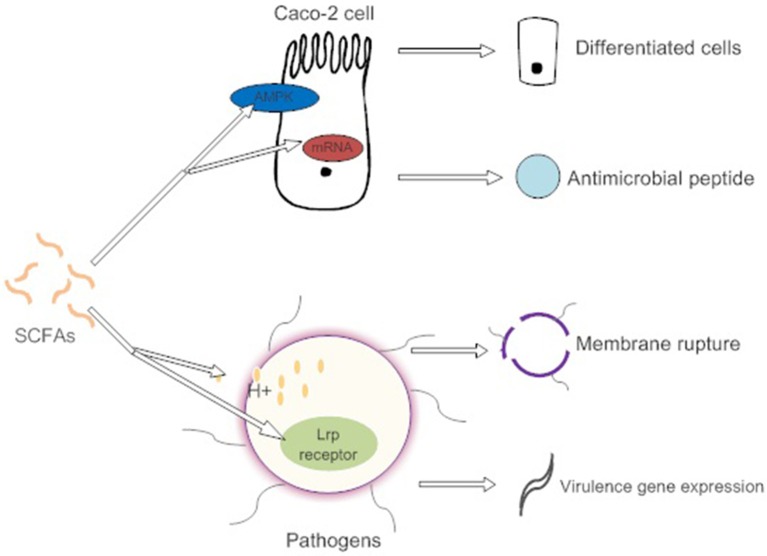
SCFAs promote the differentiation of intestinal epithelial cells by activating AMPK to adapt to environmental changes caused by pathogens. They also induce intestinal cells to synthesize antimicrobial peptides. High concentrations of SCFAs affect the osmotic pressure of pathogens and cause cell lysis. However, low concentration of SCFAs induce the expression of pathogen virulence genes.

SCFAs indirectly affect pathogen infection by maintaining the integrity of the gut barrier and activating intestinal immunity (Figure 2). For example, high concentrations of butyrate protect Caco-2 cells from *Campylobacter jejuni* invasion and translocation by inducing cell differentiation (Van Deun et al., 2008). Butyrate also increases the intestinal barrier by activating AMP-activated protein kinase (AMPK) in Caco-2 cell monolayers (Peng et al., 2009). AMPK plays a critical role in cellular energy homeostasis and performs cytoprotection under stress (Hardie et al., 2000). SCFAs (propionic acids) decrease the internalization of *S. aureus* into bovine mammary epithelial cells (bMEC), more importantly, governing the expression of antimicrobial peptide mRNA. However, the explicit relationship between membrane protein activation and cell differentiation is not clear (Alva-Murillo et al., 2012).

Direct antibacterial mechanisms of SCFAs show toxic effects on enteric bacteria and present more conspicuously at high concentrations (Van Immerseel et al., 2004). The nonionized forms of SCFAs with a circumneutral pH enter and dissociate in the bacterial cytoplasm, causing the release of SCFA anions (Lambert and Stratford, 1999). These SCFA anions affect the osmotic balance in a high concentration (Roe et al., 1998). However, low concentrations of butyrate promote the expression of the virulence determinants of *enterohemorrhagic E. coli* (EHEC) strain Sakai. The butyrate is combined with the transcriptional regulator Lrp's ligand-binding domain in response to the expression of the virulence gene (Nakanishi et al., 2009). Different spatial gradients of SCFAs act as a cue, permitting *C. jejuni* to locate the lower intestine and regulate the expression of virulence and commensalism genes (Luethy et al., 2017).

Consequently, SCFAs enhance the integrity of intestinal epithelial cells and destruct the cellular structure by influencing the osmotic pressure to affect pathogen infection. Appropriate levels of SCFAs contribute to the integrity of epithelial cells and activate the immune system, while high concentrations are toxic (Argenzio and Meuten, 1991). Therefore, the effects of SCFAs on pathogens is concentration-dependent. In the physiological range, whether SCFAs have a specific regulatory effect on epithelial homeostasis, such as affecting epithelial turnover, differentiation, or the barrier function, has not been convincingly demonstrated in humans and animals. Future studies should determine the normal physiological range of the SCFAs in the intestine and the optimal level in animal models (Gill et al., 2018).

### Microbial amino acids

Most of the amino acids in intestines originate from the metabolism of dietary proteins and tissue proteins of host or the conversion of other nitrogenous substances, while a small proportion of amino acids are synthesized *de novo* by the gut microbiota (Metges, 2000). For example, a study revealed that approximately 2–20% of circulating lysine in the body's plasma, urine, and body proteins originates from the gut microbiota, by using isotope tracers in humans, pigs, and rats (Metges et al., 2003). A few bacteria can make all 20 of the standard amino acids, which contribute to host amino acid homeostasis (Peng and Harper, 1970; Lin et al., 2017).

Bacteria synthesize a largely different set of D-amino acids (D-aa) that are released as free D-aa. A recent work has demonstrated that intestinal microbiota in mice can produce abundant free D-aas *in vivo*. Scientists quantified the D- and L-forms of all proteinogenic amino acids by two-dimensional HPLC8 in cecal contents of specific pathogen-free (SPF) mice and germ-free (GF) mice. SPF mice contained D-Ala, D-Asp, D-Glu, and D-Pro (~200–500 nmol/g) while low levels of D-Asp were only detected in GF mice. This result suggests that gut microbiota plays a vital role in producing D-aas (Sasabe et al., 2016). D-aas are involved in the composition of bacterial cell walls, and D-Ala and D-Glu are the basic constituents of bacterial peptidoglycans (Caparrós et al., 1992). However, excess D-aas (such as D-Met, D-Trp, or D-Phe) replace the D-Ala within the short peptide of peptidoglycan and cause the accumulation of two major new muropeptides, which affect the metabolism and hinder cross-linking of peptidoglycan (Caparrós et al., 1992). This means that exogenous D-aas can modulate pathogens by affecting the structure of peptidoglycan and the formation of cell walls. Moreover, D-aas also affect biofilm formation and degradation. D-Tyr (3 μM), D-Met (2 mM), D-Trp (5 mM), and D-Leu (8.5 mM) were effective in inhibiting biofilm formation of *B. subtilis*, and the synergies of all four D-amino acids were particularly potent (~10 nM). Other D-aas were inert in this experiment (Kolodkin-Gal et al., 2010). Another study observed that only D-Tyr, D-Pro, and D-Phe can inhibit the formation of *S. aureus* biofilms (Hochbaum et al., 2011). Although their action mechanism is partially associated with the replacement of D-Ala in the peptide side chain, we do not know why only a part of D-aas has the ability to regulate biofilms and why inhibiting different pathogens' biofilms require different D-aas.

In addition, D-aas are employed against gut pathogens through another pathway. D-amino acid oxidase (DAO) exists in the intestinal epithelium of mice and in human goblet cells and is secreted into the intestine lumen. The amount of DAO in SPF mice is higher than that in GE mice, indicating that gut microbiota induce the production of intestinal DAO. DAO can deaminate neutral D-aas to release the antimicrobial product H_2_O_2_. Treating some enteric pathogens (*Vibrio cholera, P. aeruginosa, S. aureus, and enterohemorrhagic E. coli*) with DAO and D-aas can inhibit their growth (Ridler, 2016). Besides, D-Ser activates the SOS response and inhibits the expression of the type III secretion system that is essential for EHEC adhering to host cells (Connolly et al., 2016). Finding commensal bacteria that produce a large amount of D-aas in gut microbiota and then examining their ability and mechanisms against pathogens *in vivo* are probably good directions (Aliashkevich et al., 2018).

### Intestinal microbial vitamins

Vitamins are essential micronutrients that maintain the normal development and health of the body. They are abundant in the human body and can be obtained through food intake or transformation and synthesis of other substances. They can also be supplied by intestinal bacteria. For example, the human intestinal bacteria LAB and *Bifidobacteria* can synthesize most of the water-soluble B vitamins and vitamin K2 from scratch (Hill, 1997). Vitamins produced by microorganisms are mainly absorbed in the colon, whereas vitamins derived from diets and hosts are taken up in the proximal small intestine (Ichihashi et al., 1992; Said and Mohammed, 2011).

*E. coli*, LAB, and *Bacteroides* species are major producers of VK2 (menaquinone) in the human body. The potential pathogenic bacteria *S. aureus* and *Salmonella* also produce VK2 (Conly and Stein, 1992; Conly et al., 1994). VK2 play an indispensable role in the virulence and survival of some pathogens. For example, *S. aureus* acquires iron through the high-affinity heme uptake system during invasion into hosts, but excessive heme is highly toxic. Membrane-based quinone molecules and heme form a redox cycle that continuously releases semiquinones and reduces heme (Wakeman et al., 2012). In addition, VK2 have been reported to affect the phenotype expression of pathogens. Different concentrations of VK2 enhance the biofilm formation of *S. aureus*, and the response to exogenous VK2 signals appears to be under the regulation of sarA in *S. aureus* (Kirby et al., 2014). In these processes, VK2 play a vital role in maintaining the survival strategies of pathogens. However, the analogs of VK2, such as menadione, 1, 4-naphthoquinone, and coenzymes Q1 to Q3, broadly inhibit the growth of pathogens and reduce the amount of exotoxin generated by gram-positive organisms. Moreover, VK2 not only is a part of the electron transport chain in *S. aureus*, but also a precursor of menaquinone (Schlievert et al., 2013).

The B vitamins [VB2(riboflavin), VB6(pyridoxine) and VB9(folacin)] are all synthesized by bacteria in the gut (LeBlanc et al., 2013). VB2 is a precursor of the coenzyme flavomononucleotide (FMN) and flavin adenine dinucleotides (FAD), and it participates in cell metabolism. LAB, *E. coli*, and *B. subtilis* combine guanosine triphosphate (GTP) and D-ribulose 5-phosphate to synthesize riboflavin (Bacher et al., 2000; Sonenshein et al., 2002). *In vivo*, an intramuscular injection of VB2 (6.25 mg/kg−100 mg/kg) can enhance the nonspecific resistance of mice to various pathogens. The effect was induced by the stimulation of the proliferation of neutrophils, monocytes, and activated macrophages. This provides evidence that VB2 indirectly regulates immune responses to affect pathogens (Araki et al., 2008). Deficient mice have impaired VB2-dependent NADPH oxidase 2(Nox2) activation and ultimately reduced defense against *L. monocytogenes* (Schramm et al., 2014). However, VB2 has different effects on pathogens *in vitro*. It has been reported that VB2 is toxic and can enhance the degree of pathogen infection at high concentrations (Flieger et al., 2016). The reasons for the diverse effects of VB2 on pathogens are still unclear, but concentration seems to be an important factor because VB2 concentration and application conditions were different in these experiments. In addition, a recent study revealed that if worms' diets contain live bacteria, they develop normally until adults. If only killed bacteria were fed, the worms stop eating and growing. Supplementing extra VB2 leads to a return to normal consumption, suggesting that intestinal microbiota plays an important role in supplying micronutrients such as VB2 (Qi et al., 2017).

Most of the members of the vitamin B family mainly induce the immune system to prevent pathogen infection. For example, VB9 is mainly produced by the fermentation of probiotics such as *bifidobacteria* and *lactobacilli* in the human body. VB9 deficiency reduces lymphocyte proliferation and natural killer cell activity, and it plays a pivotal role in the survival of regulatory T (Treg) cells (Courtemanche et al., 2004; Troen et al., 2006; Kunisawa et al., 2012). *Bacillus, E. coli, P. aeruginosa*, and *Serratia marcescens* can produce VB6 (Dempsey, 1967). The deficiency of VB6 impairs both cellular and humoral immunity in humans and animals. Supplementation with *Bacteroides* and VB6 can enhance the clearance of pathogens during *Salmonella typhimurium* infection. We noticed that VB6 can clear pathogens by promoting the growth of *Bacteroides*, regulating the host immune system, or interfering with the growth or the virulence expression of *S. typhimurium*. (Talbott et al., 1987; Rall and Meydani, 1993; Sperandio, 2017).

In summary, vitamins produced by gut microbiota play an important role in activating intestinal immune response and defending against the infection by external pathogens. However, the mechanism of direct interaction between microbial vitamins and pathogens remains unclear.

### Quorum-sensing autoinducers (AI)

With the gradual increase and accumulation of bacterial density in a certain space, the bacteria show some physiological or biochemical changes, facilitating quorum sensing (QS) (Waters and Bassler, 2005). Since the concept was proposed by Fuqua a large number of studies have found and revealed the primary mechanism and fractional functions of QS. In a QS system, one or more signaling molecules produced by bacteria are released into the cytoplasm to sense the bacterial concentration. The signaling molecules bind to QS receptors on the surface of bacteria or other regulated bacteria when the bacterial concentrations reach a certain threshold. Then, the receptors bind the corresponding binding domain of genes to change the bacterial behavior (Papenfort and Bassler, 2016).

In the gut, *E. coli* is regulated by at least three QS signaling molecules (Walters and Sperandio, 2006). One of them is *E. coli*-generated autoinducer-2 (AI-2), which is composed of 4,5-dihydroxy-2,3-pentanedione, a metabolite of S-adenosylmethionine (Pereira et al., 2013). AI-2 is encoded by the luxS gene and has diverse effects on different bacteria. For example, the luxS deletion mutant of *E. coli* reduced the adhesion of flagellum, expression of Stx2e toxin, and production of bacterial surface outer membrane proteins (Yang et al., 2014). However, a recent work demonstrated that the inactivation of the luxS gene of *S. aureus* increased biofilm formation and higher polysaccharide intercellular adhesion (PIA) production (Ma et al., 2017). Another signaling molecule is acylated homoserine lactone (AHL), mainly found in gram-negative bacteria. Although *E. coli* cannot synthesize AHLs due to the lack of the luxS gene, it has the AHL receptor protein LuxR. The AHLs produced by *P. aeruginosa* isolated from the bovine intestine and produced by *Clostridium perfringens* in the porcine intestine modulate the expression of flagella, a virulence factor of F18 *E. coli* (Yang et al., 2013). Currently, other autoinducers have also been discovered and most of them affect themselves and other pathogens. For example, 2-heptyl-3-hydroxy-4(1H)-quinolone (PQS) is a QS signaling molecule present in *P. aeruginosa*. PQS modulates various target genes including those coding for rhamnolipid, elastase, the PA-IL lectin, and pyocyanin as well as influencing biofilm formation and cellular fitness (Diggle et al., 2006). Most of the studies on QS have focused on the effects and mechanisms of pathogen virulence and the discovery of new inhibitors of QS. However, the vast majority of gut microbes are beneficial to the body. Whether QS can be used to enhance the survival ability of these probiotics and to resist pathogen invasion is still an unexplored topic in this field.

## The effects of metabolites from hosts on pathogens

Host-derived metabolites are predominant in the body. They are mainly synthesized by the host and provided by the diets. Here, we briefly summarize some of the most common host-derived metabolites' regulatory mechanisms and effects on pathogens and discuss the problems that exist. (Table 3).

**Table 3:** The pathogens are regulated by host-derived metabolites and their mechanisms and effects.

**Metabolites**		**Pathogens by regulated**	**Mechanisms and effects**
FFA		Potential pathogenic or opportunistic bacteria	Bind to the carriers of the electron transport chain or insert into the inner membrane, increase the permeability of the cell membrane. Cell death. ↓ Inhibit the activity of cell membrane enzymes and the absorption of nutrients to kill bacteria. ↓
	Linoleic acid, Arachidonic acid	*S. aureus*	Activate EsxA expression, one of the virulence proteins of T7SS. ↑
	C9, T11-CLA		Inhibit NF-κB activation and IL-12 production. ↑
	Arachidonic acid		Enhance immune response (both cellular and humoral immunity) and modulate macrophages to increase the free radicals. ↓
Amino acids	L-glutamine		Reduce the production of s-IgA and enhance the mucosal immune system. ↓
			Activate NF-κB pathway. ↓
		*L. monocytogenes*	Indicate environmental change and virulence gene induction. ↑
Vitamins	Vitamin A		Regulate immune response, such as lymphocyte proliferation, cytokine expression, and antibody production. ↓
			Maintain the normal proportion of gut microbiota. ↓
		*S. aureus* and *Mycobacteria*	Destruct bacterial biofilm formation by inhibiting EPS production and QS. ↓
	Vitamin C	*Citrobacter*	Enhance pathogenicity of harmful bacteria. ↑
	Vitamin D		Increase defensins. ↓

*↓, Inhibit pathogens, ↑, Promote pathogens*.

### Host-derived free fatty acids

Fatty acids released from host lipids by enzyme action become free fatty acids (FFAs), which play unique roles in host defenses against potential pathogenic microorganisms.

It is unclear how FFAs exert their antibacterial effects, but we know that the central target seems to be the bacterial cell membrane and the diverse essential processes that occur within and at the membrane (Desbois and Smith, 2010). For example, unsaturated FFAs probably bind to the carriers of the electron transport chain directly or insert into the inner membrane when they cross the cell wall or outer membrane of bacteria, leading to the electron carriers moving apart or being displaced from the membrane. These processes suggest that FFAs can affect the energy metabolism of bacteria by destroying the electron transfer chain. More importantly, the insertion of FFAs into the cell membrane increases the permeability of the cell membrane, resulting in the leakage of some cytoplasmic contents and eventually cell death (Galbraith and Miller, 1973; Peters and Chin, 2003; Desbois and Smith, 2010). In addition, FFAs suppress the activity of cell membrane enzymes and the nutrients absorption of bacteria (Galbraith and Miller, 1973; Shibasaki, 1978; Kurihara et al., 1999; Zheng et al., 2005; Sado-Kamdem et al., 2009). However, the type VII secretion system (T7SS) of *S. aureus* can be triggered by host-specific cis-fatty acids (Lopez et al., 2017). T7SS is a major secretory pathway for *S. aureus* to produce virulence factors during host infection, contributing to long-term persistence of *staphylococcal* survival and the formation of abscesses (Kneuper et al., 2014). A lipidomics analysis showed that the host-specific cis-unsaturated fatty acids were linoleic acid (LA) and arachidonic acid. *S. aureus* use fatty acid kinase (FAK) to integrate the fatty acids into their own phospholipids and lipoproteins, thereby activating EsxA expression, one of the virulence proteins of T7SS (Burts et al., 2005; Parsons et al., 2014; Lopez et al., 2017). We do not know why FFAs have different effects on the same pathogens. We assume that FFAs may be required in the process of pathogen growth and infection. When bacterial growth reaches a critical value, pathogens will switch to a state where a large amount of FFAs in the body is highly toxic for pathogens.

Immune responses induced by FFAs is another important regulatory mechanism. Conjugated linoleic acid (CLA) is a generic term for the position and geometric isomers of linoleic acid with conjugated double bonds at the 6-8 to 13-15 carbon positions (Bergamo et al., 2014). The cis-9, trans-11 isomer (c9, t11-CLA) of CLA inhibits nuclear factor-κB (NF-κB) activation and IL-12 production in dendritic cells through ERK-mediated IL-10 induction, suggesting that CLA affects the occurrence of infectious inflammation in the body by modulating the immune responses (Loscher et al., 2005). Arachidonic acid also simultaneously enhances immune responses (both cellular and humoral immunity). It can modulate macrophages to increase the free radicals that are critical for antimicrobial or tumor-killing effect. Macrophages and T cells in the gut can deliver arachidonic acid to target organs to eliminate pathogen infection and suppress inflammation (Das, 2018).

It is evident that FFAs can influence pathogen infection through different pathways. Therefore, they have potential value as an antimicrobial agent. FFAs are able to selectively improve the growth of beneficial bacteria and possibly prevent the proliferation of harmful microbiota; furthermore, dietary supplementation does not lead to any deleterious effect on gut microbiota (Das, 2018).

### Host-derived amino acids

Host-derived amino acids are one of the most common metabolites in the gut and the main oxidative fuel in the intestinal mucosa, as well as the structures that synthesize tissue proteins, nitrogen sources, enzymes, and other biological products. Recent studies have shown that specific amino acids (glutamine, glutamic acid, arginine, glycine, lysine, threonine, and sulfur-containing amino acids) can be used to prevent and treat gut-related diseases in humans and mammals (Wang et al., 2009; Ridler, 2016). For example, L-glutamine is a conditionally essential amino acid in mammalian plasma and an important donor of biosynthetic nucleic acids and other amino acids. It plays a major role in promoting the repair of a damaged gut, preventing pathogen infection, and maintaining the local immune function (Souba et al., 1990; Scheppach et al., 1996). L-glutamine is predominantly present in the small intestine whose intestinal epithelial barrier can protect the internal environment from the invasion of pathogens (Ménard et al., 2010; Veldhoen and Brucklacher-Waldert, 2012). The normal intestinal barrier against pathogen infection primarily depends on the specific IgA antibodies secreted by the gut-associated lymphoid tissue (GALT), which includes lymph nodes, plasma cells, and mucosal intraepithelial lymphocytes (Ruth and Field, 2013). In normal physiological conditions, IgAs are released into the intestine and can be employed as an inhibitor of bacterial adhesion. However, an abnormal regulation of secretory IgA (s-IgA) production leads to bacterial translocation and defective barrier integrity through the weakening the mucosal immune system (Mestecky et al., 1986; Artis, 2008). The addition of L-glutamine reduces the production of s-IgA, helping the intestinal immune system to resist pathogen adhesion and colonization (Alverdy, 1990; Wang et al., 2009). Besides, L-glutamine can activate NF-κB signaling pathway when pathogens invade into the host. This pathway is an essential transcription factors family in the intestinal cells and produces a variety of cytokines to regulate the immune response (Haynes et al., 2009; Pasparakis, 2009; Mondello et al., 2010).

However, amino acids act as a double-edged sword for gut health that not only contribute to disease prevention but also induce the expression of pathogenic virulence genes. L-glutamine can serve as an indicator of environmental change and expression of virulence genes in *L. monocytogenes*. The intestinal exogenous L-glutamine crosses the bacterial membrane through ABC transporters and accumulates in the cytoplasm when *L. monocytogenes* invade into hosts, then, the bacteria would know that they have successfully entered host environments by sensing the concentration of exogenous L-glutamine and ultimatly release the endogenous virulence (Haber et al., 2017). Similarly, glutathione, which consists of glutamic acid, glycine, and cysteine, also acts as a signaling molecule to activate the expression of the virulence regulator PrfA in *L. monocytogenes* (Reniere et al., 2015).

### Host-derived vitamins

Host-derived vitamins are the main source of most vitamins in the intestines, hence their roles in the body are more pronounced. Vitamin A is obtained directly from the diets or in the form of retinyl esters and carotenoids. According to studies in animal models, it can be considered as an anti-infective vitamin owing to its important role in the immune system, such as for regulating lymphocyte proliferation, cytokine expression, and antibody production. The role of vitamin A is mainly associated with intestinal epithelial cells (Semba, 1999). A lack of vitamin A impairs the integrity of the intestinal mucosal barrier, changing the gut microbiota and mucus protein expression and components, reducing the immunity, increasing the risk of susceptibility to pathogens, and spontaneously inducing the death of thymocytes and mature T cells. In addition, all-trans retinoic acid, a metabolite of vitamin A in the intestinal mucosal dendritic cells, shapes the intestinal immune responses and establishes the tolerance of intestinal immunity, thereby affecting the body's specific immune function (Amit-Romach et al., 2009; Litwack and Litwack, 2011; Cassani et al., 2012). Clinical trials have also shown that supplementation of vitamin A increase the cure rate of infectious diseases (Green and Mellanby, 1928; Semba, 1999). For example, diarrhea is a common disease caused by pathogens in the intestine. Vitamin supplementation reduces the incidence and mortality of diarrheal diseases in children. Though the detailed mechanism of action is not entirely clear, it is certain that the regulation of vitamin A for the mucosal immune system may depend on the type of enteric pathogens (Long et al., 2006). Another reason perhaps is that the diversity of intestinal microbiota will decrease in the absence of vitamins. For example, there is a significant reduction in butyric acid-producing bacteria in children's gut microbiota when vitamin A is deficient, whereas the proportion of Enterococcus is significantly increased (including *Clostridium* and *Clostridium butyricum*). An appropriate level of vitamin A inhibits the overgrowth of pathogens and strengthens the defense barrier of the intestine. *Enterococci* increase the expression of inflammatory cells and destroy the mucosa of the gastrointestinal tract (Lv et al., 2016; Wang et al., 2016).

Vitamin C is a major dietary nutrient that can kill or inhibit the growth of pathogens. For example, 5 mM vitamin C completely inhibits the growth of *S. aureus* (Kallio et al., 2012; Vilchèze et al., 2013). Recently, one of the antibacterial mechanisms of vitamin C revealed that the bacterial biofilm formation was effectively destructed by inhibiting the production of extracellular polymeric substances (EPS). Vitamin C inhibits QS and other stationary phase control mechanisms that support biofilm development and specifically lead to inhibition of polysaccharide biosynthesis (Pandit et al., 2017).

Other vitamins, such as vitamin E and vitamin D, are also linked to the homeostasis of the intestinal environments. For example, in the absence of vitamin E, the pathogenicity of *Citrobacter* in the intestinal tract of mice is enhanced (Smith et al., 2011). Vitamin D deficiency reduces the number of defensins that are necessary antimicrobial molecules required to maintain the intestinal microbiota in the body (Su et al., 2016). In summary, vitamin deficiency can affect the structure of the gut microbiota. The mechanism of the action of vitamins on pathogens is no different from that of other metabolites that have been described above, mainly inhibiting the developments of pathogens and activating the immune system.

## Conclusion and direction

Intestinal microbiota and their metabolites, as a whole, are now considered an important and versatile “organ” that maintains the body's physiological homeostasis. Application of the metabonomics technology, isotope tracer method, and fluorescent probe technology has led to numerous evidences suggesting that metabolites play a vital role in regulating the infection of pathogens. On the one hand, these metabolites prevent the invasion of pathogens by killing the pathogens directly, resisting the colonization and internalization, and inducing the immune responses indirectly. On the other hand, metabolites are also involved in the process that contributes to the pathogen infection. Some metabolites serve as signaling molecules to activate bacterial quorum sensing that allows bacteria to produce toxins and form biofilms, and to promote the invasion of pathogens by activating the expression of virulence factors. More importantly, we found that these mechanisms also share common characteristics. The interaction between metabolites and the membrane of pathogens is of great significance. Secondly, metabolites also activate a large number of immune factors to defend against pathogens. These two common characteristics may provide ideas for further exploring the regulation of metabolites on pathogens. Moreover, we also found some problems in these studies. The reasons for the diverse effects of some metabolites on pathogens are still unclear, and we speculate that this is related to the different growth stages of pathogens.

Therefore, we provide some possible directions for future research. First, we should further explore the regulation mechanisms of various metabolites on pathogens, especially the interaction with bacterial membranes and the induced immune response in the intestines. Changes in the levels of genes involved in regulation of pathogens by metabolites should also be noted (Qin et al., 2018). Second, metabolomics should be used to quantitatively analyze the amounts of various metabolites in animal models, while developing novel specific fluorescent probes for different metabolites to track and localize metabolites from different sources in the gut. Finally, we hope that we will relieve or eliminate pathogens by inducing specific metabolites from specific gut bacteria and hosts in the future.

## Author contributions

GZ, WR, and ZL designed the review article, and ZL wrote the review article. GQ, XD, and PH revised the review article. XJ, DZ, and XW helped with good discussions and comments and provided relevant literature. YY conducted a detailed examination of the content, structure and citation of the article.

### Conflict of interest statement

The authors declare that the research was conducted in the absence of any commercial or financial relationships that could be construed as a potential conflict of interest.
